# Dietary Encapsulated Essential Oils Improve Production Performance of Coccidiosis-Vaccine-Challenged Broiler Chickens

**DOI:** 10.3390/ani10030481

**Published:** 2020-03-13

**Authors:** Jeong-Woo Lee, Da-Hye Kim, Yoo-Bhin Kim, Su-Been Jeong, Sung-Taek Oh, Seung-Yeol Cho, Kyung-Woo Lee

**Affiliations:** 1 Department of Animal Science and Technology, Sanghuh College of Life Sciences, Konkuk University, 120 Neungdong-ro, Gwangjin-gu, Seoul 05029, Korea; dlwjddn0123@naver.com (J.-W.L.); kdh142536@naver.com (D.-H.K.); ybin51@naver.com (Y.-B.K.); jsbjsy317@naver.com (S.-B.J.);; 2 EUGENE BIO, Suwon-si, Gyeonggi-do 16675, Korea; eugenebio@naver.com; 3 Animal Biosciences and Biotechnology Laboratory, Beltsville Agricultural Research Center, Agricultural Research Service, US Department of Agriculture, Beltsville, MD 20705, USA

**Keywords:** encapsulated essential oils, coccidiosis, growth performance, broiler chickens, gut health

## Abstract

**Simple Summary:**

The in-feed antibiotics have been banned worldwide, and anticoccidial drugs are also expected to be removed from the formulated, complete feeds. Thus, looking for alternatives to anticoccidials has been on the increase. Essential oils are naturally derived substances containing the aromatic components of herbs and spices and exhibit antibacterial/anticoccidial, antioxidant, and immune modulating-effects, the properties in poultry. These beneficial biological properties of essential oils make them be considered potential anticoccidial agents. Forthermore, encapsulating essential oils is known to be an effective and efficient strategy to slowly release their active components upon passing the gastrointestinal tract. This study was conducted to examine the effects of encapsulated thymol- and carvacrol-based essential oils on productivity and gut health of chickens challenged with high dose of coccidiosis vaccine.

**Abstract:**

The present study was conducted to evaluate the encapsulated essential oils (EEO) as an alternative to anticoccidials using a coccidiosis vaccine challenged model in broiler chickens. A total of 600 one-day-old male broiler chicks were provided with no added corn/soybean-meal-based control diet or diets that contained either salinomycin (SAL) or thymol- and carvacrol-based EEO at 60 and 120 mg per kg of diet. Before challenge at 21 days, each treatment had 10 replicates except for the no-added control group, which had 20 replicates. On day 21, half of the control groups were orally challenged with a coccidiosis vaccine at 25 times higher than the recommended vaccine dose. During 22 to 28 days (i.e., one-week post coccidiosis vaccine challenge), the challenged chickens had a decrease (*P* < 0.05) in body weight gain and feed intake but an increase in feed conversion ratio compared with the non-challenged, naïve control chickens. However, dietary EEO significantly counteracted (*P* < 0.05) coccidiosis-vaccine-induced depression in body weight gain and feed intake. Inclusion of dietary EEO linearly decreased (*P* < 0.05) the concentrations of the volatile fatty acids. Dietary SAL and EEO affected gut morphology in chickens at 20 days post-hatch. Dietary EEO linearly (*P* = 0.073) increased serum catalase activity as the inclusion level increased. Collectively, our study shows that dietary EEO increased coccidiosis-vaccine-induced growth depression and altered gut physiology in broiler chickens. Our study adds to the accumulating evidence that dietary EEO is proven to be an effective alternative to anticoccidials for broiler chickens.

## 1. Introduction

Avian coccidiosis is caused by several species of *Eimeria*, which are ubiquitous in nature and infectious protozoa that penetrate and damage the epithelial cells of intestinal tissue, resulting in intestinal inflammation and hemorrhage [1]. The intestinal damage decreases feed intake (FI), retards growth, and suppresses humoral and cell-mediated immune responses, all of which lead to significant adverse implications for the commercial poultry industry [2]. The annual financial losses by coccidiosis in the poultry industry worldwide have been estimated at US $3 billion, which is mainly due to use of prophylactic or therapeutic in-feed medications and compromised health status of afflicted chickens [3].

It is well-known that the presence of *Eimeria* spp. is often found in various environments including used litters or chick delivery boxes. The *Eimeria*-affected flocks are characterized by the presence of multiple species of *Eimeria* affecting sites from duodenum to ceca as they have gut-site-specific infectivity [3]. In addition, *Eimera* spp., once infected, have inherent mechanisms to evade the host immunity [1,2]. Until now, subtherapeutic anticoccidials including ionophores (e.g., salinomycin) have been used to prevent avian coccidiosis. However, there have been increasing concerns on the occurrence of drug-resistant oocytes and the drug residues in broiler meats. As antibiotic-free broiler production has been in practice with legislative or voluntary ban on in-feed antibiotics, in-feed anticoccidials are expected to be removed from the diets of chickens. Thus, global poultry industry is urged to develop alternatives to anticoccidials for sustainable antimicrobial-free poultry production. 

In response to increasing global needs for alternatives to anticoccidials, nutrition-based strategies have been implemented to control avian coccidiosis caused by the *Eimeria* spp. [4]. Among the potential candidates as alternatives, the plant-derived essential oils (EO) have been explored as they exhibit various biological properties, including antimicrobials, antioxidants, and immune-modulation [5]. Among the EOs studied, thymol and carvacrol are the major components of thyme or oregano EOs and are likely to have similar mechanisms of antimicrobial activity [6]. In addition, [7] addressed that dietary EO increased the productivity of the *Eimeria*-infected broiler chickens. Due to the chemical nature of EOs and their components having low molecular weights, they are rapidly absorbed in the upper segments of intestine upon ingestion and known to directly or indirectly affect intestinal microflora and secretion of endogenous digestive enzymes [5,8]. In order to maximize the effect of EO as an anticoccidial agent, it is necessary to reach the infected area proximally to distally. Therefore, it is expected that the anticoccidial effect of EO will be enhanced if they are technically encapsulated to release active components slowly during passage of the gut, thus enabling action on the *Eimeria* present in duodenum to ceca. Similar strategies with encapsulated EO (EEO) on necrotic enteritis or Salmonella have been reported [9,10].

The purpose of this study was to investigate the effects of thymol- and carvacrol-based EEO on the productivity and gut health of broiler chicks inoculated with high doses of coccidiosis vaccine. In this study, we used live coccidiosis vaccine to induce experimental coccidiosis as documented elsewhere [11,12,13]. Earlier conflicting reports that *Eimeria* infection decreased rectal temperature [14] or dietary EEO relieved heat-stress chickens [15,16] brought us to measure the body surface temperature of the challenged chickens.

## 2. Materials and Methods

### 2.1. Animal Care

The experimental procedure was approved by the Institutional Animal Care and Use Committee of Konkuk University (KU18095).

### 2.2. Experimental Design, Animals and Diets

A total of 600 1-day-old feather-sexed male broiler chicks (Ross 308) were obtained from a local hatchery. Upon arrival, they were individually weighed and randomly placed into 50 floor pens (1 m × 2 m). The chicken facility was initially set at 32 °C, was gradually decreased to 25 °C at 3 weeks, and then kept constant thereafter. The light was set with one-hour darkness per day. The windowless chicken facility was thoroughly disinfected before the experiment, and fresh rice husks as a bedding material were used.

A corn and soybean meal-based diet was used as a control diet (Table 1), and the experimental diets were formulated by mixing the control diet with salinomycin (60 mg/kg) (SAL) or EEO preparations at the levels of 60 and 120 mg/kg of diet. The EEO preparations used contained an equal concentration of thymol and carvacrol at the level of 140 g per kg of preparation as active components and were microencapsulated (Vetagro SpA, Italy) to prevent loss during the pelleting process and/or to allow slow release upon ingestion to reach to the distal intestine (e.g., cecum). It is reported that EEO added into mash or pellet diets are stable and able to release its active component throughout the intestine [17]. This EEO preparation used in this study is currently marketed as the natural alternative to anticoccidials (EUGENE BIO Co., Gyeonggi-do) in South Korea.

Day-old chicks were provided with either control or experimental diets from the beginning. Each treatment had 10 replicates of 12 chicks each (*n* = 120 chicks/treatment) except for the control group, which had 20 replicates. At 21 days, half of the control groups (n = 10 replicates/treatment) and all experimental groups were orally gavaged with 25× the recommended dose to induce coccidiosis [11,12,13]. Chickens not inoculated with coccidiosis vaccine were gavaged with phosphate-buffered saline and considered the nonchallenged, naïve control groups (*n* = 10 replicates/treatment). Feed intake and body weight per pen were measured weekly. Mortality was recorded when it occurred and was used to calculate mortality-adjusted feed conversion ratio.

### 2.3. Sampling

At 21 and 28 days, 1 bird per pen was randomly selected and euthanized by overdose of CO_2_ gas. At 21 days, immediately after euthanasia, the small intestine was excised and sampled for the measurement of gut morphology. At 28 days post-hatch (i.e., one week post coccidiosis vaccine challenge), blood was collected into vacutainer tubes by heart puncture immediately after euthanasia. Serum samples were obtained by gentle centrifugation 200 × *g* for 15 min and stored at −20 °C until use. Immediately after blood sampling, small intestine was sampled for counting *Eimeria*-specific lesion scores and a pair of ceca were excised for measurement of volatile fatty acids. In addition, at 28 days, two birds per pen were randomly selected to record the chicken’s surface temperature (FLIR-300 Infrared Camera). 

### 2.4. Gut Morphology

Midsections (approximately 1-cm-long segment) of duodenum, jejunum, and ileum sampled at 21 days were fixed in 10% neutral-buffered formalin for a minimum of 48 h, and 4.0 µm sections were prepared. The sections were dyed with standard hematoxylin-eosin solution. The villus height (VH) was measured from the villus tip to the villus bottom. The crypt depth (CD) was defined from villus bottom to the crypt. The ratio of villus height and crypt depth (VH:CD) was then calculated.

### 2.5. Body Surface Temperature Index

On one-week (i.e., 28 days post-hatch) post coccidiosis vaccine challenge, two birds were selected for measuring the surface temperature. The surface temperature of the body was measured by taking a head of the broiler, a breast (abdomen), and a leg portion using a thermally sensed image cam (FLIR-300).

### 2.6. Lesion Score

Approximately 20-cm-long mid-segments of duodenum and jejunum sampled at 28 days (i.e., one-week post vaccine infection) were taken and cut longitudinally. Intestinal contents were gently removed and lesion scores from 0 to 4 in ascending order of severity as described elsewhere [18] were independently made by 3 observers in a blinded fashion with no knowledge of treatment groups.

### 2.7. Measurement of Volatile Fatty Acids

Approximately 1 g of cecal content sampled at 28 days was homogenized with 0.05 ml of saturated solution HgCl_2_, 1 ml of 25% H_3_PO_4_, and 0.2 ml of 2% pivalic acid as an internal standard and centrifuged. Then, the supernatant was collected and stored at −20 °C before analysis. Volatile fatty acids (VFA) were measured using gas chromatography (6890 Series GC System, HP, Palo Alto, CA, USA) as described elsewhere [19]. The temperature of the inlet oven and detector were set at 220 °C, 100 °C, and 250 °C, respectively. Each sample for VFA analysis was duplicated. 

### 2.8. Measurement of Biochemical and Antioxidant Parameters in Serum Samples

Serum samples collected at 28 days were analyzed for glutamic-oxaloacetic transaminase, glutamic-pyruvic transaminase, triglyceride, total cholesterol, and uric acid using an automatic dry biochemical analyzer (Film DRI CHEM 7000i, Fuji film, Tokyo, Japan). The concentrations of NO in serum samples were determined as described elsewhere. NO concentration was calculated from standard curve with sodium titrate as described [20,21]. For antioxidant markers in serum samples, malondialdehyde contents using TBARS assay kit (OxiSelect^TM^ TBARS Assay Kit-MDA Quantitation, Cell Biolabs Inc., San Diego, CA, USA), catalase (OxiSelect^TM^ Catalase Activity Assay kit, Cell Biolabs Inc., San Diego, CA, USA), superoxide dismutase (SOD) (SOD determination assay kit-WST, Sigma, St. Louis, MO, USA), and total antioxidant capacity (TAC) (QuantiChrom^TM^ antioxidant assay kit-DTAC 100, BioAssay Systems, Hayward, CA, USA) were measured per the manufacturers’ recommendation.

### 2.9. Statistical Analysis

The pen was considered an experimental unit. All data were evaluated by one-way analysis of variance using the general linear model (GLM) procedure of SAS 9.4 (SAS Institute Inc., Cary, NC, USA). If the F-test for treatment effect was significant, differences between treatment means were determined using Duncan’s multiple range test. In addition, orthogonal polynomial contrasts were used to assess the significance of graded EEO addition against either nonchallenged or challenged controls depending on the variables measured before or after coccidiosis vaccine challenge. The significance was preset at *P* < 0.05.

## 3. Results

### 3.1. Growth Performance

None of the dietary treatments affected body weight gain, feed intake, and feed conversion ratio during the starter phase (Table 2). Coccidiosis vaccine challenge significantly impaired body weight gain compared with the naïve control group during the finisher phase. However, chickens fed diets containing SAL or EEO increased body weight gain (*P* < 0.05) compared with the challenged control group and did not differ from the naïve control groups (Table 2). The challenged control chickens ate least (*P* = 0.087) during the finisher period, being lower by on average 11.5% compared with the nonchallenged control group and by 6.5% to 7.9% compared with the challenged/treated groups. Dietary EEO quadratically increased body weight gain (*P* = 0.002) and feed intake (*P* = 0.007) as the inclusion level increased. None of the treatments affected feed conversion ratio during the finisher phase, albeit that chickens fed EEO at 120 mg/kg of diet had the lowest feed conversion ratio.

### 3.2. Intestinal Morphology

Duodenal villus heights were significantly increased in SAL-fed chickens compared with the control group, and dietary EEO quadratically increased duodenal villus height (Table 3). On the other hand, jejunal and ileal villus heights were not affected by dietary treatments. Duodenal crypt depth was not affected by dietary treatments. Jejunal crypt depth was lowest in SAL-fed chickens and was quadratically increased with increasing dietary EEO. However, crypt depths at duodenum and ileum were not altered by dietary treatments. Duodenal villus height-to-crypt depth ratio was quadratically increased as dietary EEO increased. Its ratio at jejunum was highest in a SAL-added diet-fed chicken and quadratically decreased with increasing EEO. Dietary EEO at 120 mg/kg had highest ileal villus height: crypt depth ratio (*P* < 0.05).

### 3.3. Gut Lesion Score

Gut lesions at duodenum and jejunum were scored at 7 days post coccidiosis vaccine challenge. No *Eimeria*-specific lesion was noted in the naïve control chickens. Duodenal lesion was linearly lowered (*P* = 0.054) with increasing EEO (Table 4). Chickens fed a diet containing EEO at 60 mg/kg of diet exhibited (*P* < 0.05) highest jejunal lesion (Table 4). In general, *Eimeria*-specific gut lesions were kept low.

### 3.4. Cecal Volatile Fatty Acids

No difference in concentrations of cecal volatile fatty acids was noted between the non-challenged vs. challenged control groups (Table 5). The concentration of volatile fatty acids (i.e., acetate, valerate, branched-chain fatty acids, and total short-chain fatty acids) in cecal contents linearly decreased as the dietary EEO increased. It was noted that chickens fed a diet containing EEO at 120 mg per kg of diet had the lowest concentration (*P* < 0.05) of acetate and total short-chain fatty acids. 

### 3.5. Body Surface Temperature

In this study, broilers’ body surface temperature was measured using an infrared camera at 7 days post coccidiosis vaccine challenge (Table 6). No difference in body surface temperature at three locations between the naïve and challenged control groups was noted. On the other hand, the body surface temperatures of the head, breast, and leg were significantly lower (*P* < 0.05) in chickens fed EEO-added diets compared with the challenged-control group and linearly decreased (*P* < 0.05) with increasing dietary EEO (Table 6). On the other hand, dietary SAL failed to affect the body surface temperature. 

### 3.6. Serum Parameters

None of the serum parameters, including total cholesterol, triglycerides, glutamic-oxaloacetic transaminase, glutamic-pyruvic transaminase, uric acid, and nitric oxide, were affected by coccidiosis vaccine challenge or dietary treatments (Table 7). Serum concentrations of uric acid tended (linear effect, *P* = 0.065) to decrease with increasing dietary EEO. 

### 3.7. Antioxidant Marker Assays

Concentration of malondialdehyde in serum samples significantly increased in the challenged control vs. naïve control groups (Table 8). However, dietary SAL or EEO did not affect malondialdehyde content, although chickens fed a diet containing EEO at 60 mg per kg of diet tended to decrease it by on average 16.3% compared with the challenged control group. Coccidiosis vaccine challenge or dietary treatments did not affect the concentrations of catalase, SOD, or TAC in serum samples (Table 8). However, serum catalase activity tended to increase (linear effect, *P* = 0.073) as the dietary EEO increased.

## 4. Discussion

In this study, nonchallenged control chickens gained approximately 1876 g/bird during a 35-d feeding trial, which was lower compared with that specified by the broiler breeder performance standard. This is likely to be due to the experimental design and management employed in this study. We used a powdered diet to prepare the experimental diets and raised chickens at floor pens, which might have limited the potential performance. Indeed, in a recent trial with EO in coccidiosis-challenged chickens conducted in our laboratory, the control chickens fed a crumble/pelleted diet during starter and grower periods gained approximately 2400 g/bird during 35 days (data not shown). In addition, the litter-based floor pen with low stocking density (5 birds/m^2^) may in part have played a role in increasing the requirement for maintenance energy. 

It is clear from this study that coccidiosis vaccine overdose can be used as an alternative challenge strain to *Eimeria* field isolates [11,12,13], and dietary SAL exhibited an anticoccidial effect [22]. Addition of EEO into the diets of broilers challenged against coccidiosis vaccine mitigated *Eimeria*-vaccine-induced depression in body weight gain and feed intake without affecting feed conversion ratio. Thus, it is likely that dietary EEO mainly overcomes the negative effect of the coccidiosis vaccine challenge by increasing feed intake. In line with our study, dietary EO increased growth performance, enhanced nutrient digestion, and altered body composition in broiler chickens [23]. Furthermore, it was reported that dietary thymol or carvacrol is known to increase amino acid digestibility [24] and activities of endogenous digestive enzymes [23,25] in broilers. Finally, EO preparations including carvacrol or thymol are known to mitigate *Eimeria*-induced growth depression in chickens [26]. 

The protective effect of EO on *Eimeria*-specific lesions was reported elsewhere [27]. However, no clear effect of SAL or EEO on gut lesion scores was noted. In general, duodenal and jejunal lesions were kept low in the challenged chickens. This might be related to the live/attenuated vaccine strain used to induce avian coccidiosis in this study and/or delayed sampling which was conducted at 7 days post challenge. Thus, the reported coccidiosis lesions [13] by vaccine strain might have been weakened or partially recovered. In general, it is well-known that field isolates of *Eimeria* could induce more severe gut lesions compared with coccidiosis vaccine overdose [13]. It would need to use more vaccine doses if it is considered feasible to induce severe gut lesion scores. On the other hand, the finding that the coccidiosis vaccine overdose significantly reduced growth performance supports the feasibility of the experimental coccidiosis model using the vaccine strain. 

Villus height, crypt depth, or their ratios are considered the best indicators for the health and function of the intestine in chickens [28]. Dietary SAL increased duodenal villus height and jejunal villus height: crypt depth ratio, but decreased jejunal crypt dept compared with the control group. Our study corroborates earlier studies [29,30], which reported that dietary antimicrobials improved gut morphology in broiler chickens. Increasing dietary EEO quadratically increased duodenal villus heights, jejunal crypt depth, and duodenal villus-height-to-crypt-depth ratio, but quadratically lowered jejunal and ileal villus-height-to-crypt-depth. In line with our findings, dietary EO-based preparations are known to alter gut morphology in naïve chickens [31] or those challenged with coccidiosis [32]. Based on these findings, it can be speculated that dietary SAL and EEO might help to mitigate coccidiosis-induced deterioration in gut morphology, thus leading to better feed digestion and absorption that improved production performance in these groups. 

Volatile fatty acids are used as a nutrient source for colon epithelium cells and have an inhibitory effect on pathogenic bacteria in intestine [33]. In this study, cecal volatile fatty acids were not affected by coccidiosis vaccine challenge but linearly decreased with increasing EEO in diets. In contrast to our finding, recent studies [34,35] showed that *Eimeria* challenge altered cecal volatile fatty acids. The difference may be due to the strains used—vaccine vs. field isolates. Nonetheless, dietary EEO linearly lowered acetate, valerate, BCFA, and total SCFA in cecal contents. These EEO effects on volatile fatty acids might be related to either direct inhibitory effect on gut bacteria or indirectly mediated via enhanced nutrient digestibility or both. Earlier studies [36,37] showed that dietary thyme or oregano EO or their combinations modified gut volatile fatty acids in broiler chickens. If the direct inhibitory effect by EO on gut microflora was considered a major acting mechanism for lowered volatile fatty acids, then it is likely that the encapsulation used in this study would release or supply its active components to the distal part of the intestine [38].

As for body surface temperature, an interesting result emerged from this study. Both coccidiosis challenge and dietary SAL did not affect the body surface temperature, but dietary EEO significantly lowered surface temperature of head, breast, and leg, their effects being dose-dependent (*P* < 0.05). Our study indicates that dietary EEO may regulate or alter themo-regulation of the chicken, which can relieve the negative effect of heat stress. It has been reported that dietary peppermint or oregano EO at the level of 250 mg per kg of diet increased growth performance in broiler chickens under heat stress [15,16]. In addition, [39] reported that dietary EO alleviates the stress indicators induced by high stocking density in broiler chickens. Thus, our study and earlier studies [15,16,39] provide potential EO applications as a stress reliever in environmental stress conditions (e.g., heat and cold stress or immune compromise) in poultry production. Whether the observed effect of EEOs on body surface temperature is ascribed to the encapsulation process or to the EO per se needs to be addressed.

As to serum parameters, coccidiosis challenge or dietary EEO treatments did not affect any of the parameters, including total cholesterol, triglycerides, glutamic-oxaloacetic transaminase, glutamic-pyruvic transaminase, uric acid, and nitric oxide in serum samples. It is however noted that dietary EEO tended to linearly lower uric acid levels in broiler chickens. Whether this reduction in uric acid is related to low levels of amino acid oxidation [40] needs to be verified.

It is well reported that *Eimeria* infection disrupts oxidative balance leading to pathogenic oxidative stress in broiler chickens [41]. In this study, coccidiosis vaccine challenge increased serum concentration of malondialdehyde in broiler chickens, which supports the idea that coccidiosis induced host oxidative stress. Dietary EEO consisting of thymol and carvacrol at the level of 60 mg per kg of diet tended to lower serum malondialdehyde levels compared with that in the challenged control group (*P* < 0.05). In addition, dietary EEO linearly increased serum catalase activity, although statistical significance was not detected. Dietary EO or their combinations have been known to increase antioxidant capacities in naïve chickens or those challenged with lipopolysaccharide [42], *Clostridium perfringens* [43], *Eimeria* [26], or *Salmonella* spp. [44]. Thus, antioxidative properties of EO are considered an important factor as the alternatives to anticoccidials that may be responsible for mitigating the coccidiosis-induced growth depression, damaged gut mucosa, and altered physiological responses in broiler chickens.

## 5. Conclusions

In conclusion, dietary SAL and EEO significantly mitigated coccidiosis-vaccine-induced growth depression in broiler chickens, which was mediated by increase in feed intake. Dietary EEO clearly lowered body surface temperature, marginally improved antioxidant systems in chickens, and lowered the concentrations of volatile fatty acids as the inclusion level increased. Taken together, our study suggests that dietary EEO can be used as the alternative to anticoccidial agents to mitigate coccidiosis-induced growth depression in broiler chickens. Further studies are warranted about whether dietary EEO could counteract environmental stressors such as heat stress or stocking density and improve gut microbiome, antioxidative defense system, and gut barrier functions in broiler chickens.

## Figures and Tables

**Table 1:** Ingredients and composition of the basal diets (as-fed basis).

Ingredient (g/kg)	Starter (0–21 d)	Finisher (22–35 d)
Maize, 8.8% CP ^1^	570.0	636.5
Corn soybean meal, 44.8% CP	320	255
Corn gluten meal, 60% CP	40	40
Soybean oil	25	30
Salt	3.0	2.4
Dicalcium phosphate	17	13
DL-methionine, 99%	3.1	2.0
L-lysine, 78%	2.0	2.2
L-threonine	0.5	0.5
Limestone	13	13
Sodium bicarbonate	2.4	1.4
Choline chloride, 50%	2.0	2.0
Vitamin premix ^2^	1.0	1.0
Mineral premix ^3^	1.0	1.0
Total	1000.0	1000.0
Nutrient composition ^4^		
AMEn ^1^ (kcal/kg)	3049	3152
Dry matter, %	89.1	89.3
Crude protein, %	22.1	19.8
Lysine, %	1.26	1.10
Met + Cys, %	1.00	0.84
Calcium, %	1.00	0.9
Available phosphorus, %	0.45	0.37

^1^ CP = crude protein, AMEn = nitrogen-corrected apparent metabolizable energy. ^2^ Vitamin premix provided following nutrients per kg of diet: vitamin A, 12,000 IU; vitamin D_3,_ 3000 IU; Vitamin E, 40 IU; vitamin K_3_, 2 mg; vitamin B_1_, 2 mg; vitamin B_2_, 5 mg; vitamin B_6_, 3 mg; vitamin B_12_,0.02 mg; niacin, 40 mg; biotin, 0.15 mg; pantothenic acid, 10 mg.^3^ Mineral premix provided following nutrients per kg of diet: Fe, 88 mg; Mn, 66 mg; Zn, 60 mg; I, 0.99 mg; Se, 0.22 mg; Cu, 72.6 mg; Co, 0.33 mg. ^4^ Calculated value (as-fed basis).

**Table 2:** Effect of dietary encapsulated essential oils on growth performance in coccidiosis-vaccine-challenged broiler chickens ^1^.

Item	N CON ^3^	P CON	SAL	EO60	EO120	SEM ^5^	*P*-value		
							ANOVA	L ^4^	Q ^4^
**Before challenge (0 to 21 days post-hatch)**									
Body weight gain, g/d/bird	32.5 ^2^	-	32.5	31.6	31.4	0.49	0.179	0.141	0.371
Feed intake, g/d/bird	43.4 ^2^	-	44.2	42.2	42.4	0.80	0.238	0.251	0.134
Feed conversion ratio, g/g	1.34 ^2^	-	1.36	1.34	1.35	0.02	0.751	0.907	0.315
**After challenge (22 to 35 days post-hatch)**									
Body weight gain, g/d/bird	85.3 ^a^	76.2 ^b^	83.8 ^a^	81.8 ^a^	84.7 ^a^	1.59	0.018	0.561	0.002
Feed intake, g/d/bird	131	116	126	123	124	2.97	0.087	0.357	0.007
Feed conversion ratio, g/g	1.53	1.52	1.51	1.50	1.47	0.02	0.220	0.407	0.869

^1^ Values are least-squares means of 10 replicates unless otherwise stated. ^2^ Values are least-squares means of 20 replicates. ^3^ N CON = nonchallenged (naïve) control; P CON = challenged control; SAL = salinomycin at 60 mg/kg; EO60 = encapsulated essential oil at 60 mg/kg, EO120 = encapsulated essential oil at 120 mg/kg. ^4^ L = linear; Q = quadratic. ^5^ SEM = pooled standard errors of the means. ^abc^ Means without a common superscript letter differ (*P* < 0.05).

**Table 3:** Effect of supplementation of encapsulated essential oil on morphology of small intestine in naïve broiler chickens ^1.^

Item	N CON ^4^	P CON	SAL	EO60	EO120	SEM ^6^	*P*-value		
							ANOVA	L ^5^	Q ^5^
**Villus height, μm**									
Duodenum	1678 ^b2^	-	1884 ^a^	1794 ^ab^	1758 ^ab^	53.1	0.044	0.128	0.042
Jejunum	1541 ^2^	-	1476	1577	1252	95.0	0.063	0.746	0.454
Ileum	871 ^2^	-	836	949	922	57.6	0.440	0.255	0.238
**Crypt depth, μm**									
Duodenum	345 ^2^	-	321	350	320	12.9	0.231	0.783	0.120
Jejunum	225 ^a2^	-	185 ^b^	243 ^a^	222 ^a^	12.6	0.014	0.213	0.002
Ileum	193 ^2^	-	182	210	174	12.2	0.178	0.236	0.174
**VH: CD ^3^ ratio, μm: μm**									
Duodenum	5.00 ^2^	-	6.01	5.14	5.56	0.29	0.067	0.742	0.018
Jejunum	6.98 ^ab2^	-	8.20 ^a^	6.52 ^b^	5.73 ^b^	0.48	0.007	0.426	0.013
Ileum	4.55 ^b2^	-	4.39 ^b^	4.31 ^b^	5.49 ^a^	0.19	0.001	0.315	0.854

^1^ Values are least-squares means of 10 replicates unless otherwise stated. ^2^ Values are least-squares means of 20 replicates. ^3^ VH: CD ratio = villus height to crypt depth ratio. ^4^ N CON = non-challenged (naïve) control; P CON = challenged control; SAL = salinomycin at 60 mg/kg; EO60 = encapsulated essential oil at 60 mg/kg, EO120 = encapsulated essential oil at 120 mg/kg. ^5^ L = linear; Q = quadratic. ^6^ SEM = pooled standard errors of the means. ^abc^ Means without a common superscript letter differ (*P* < 0.05).

**Table 4:** Effect of dietary encapsulated essential oils on gut lesion scores in coccidiosis-vaccine-challenged broiler chickens^1.^

Item	N CON ^2^	P CON	SAL	EO60	EO120	SEM ^4^	*P*-value		
							ANOVA	L ^3^	Q ^3^
**28 days on lesion score**									
Duodenum	0.00	0.48	0.50	0.60	0.21	0.18	0.133	0.054	0.312
Jejunum	0.00 ^b^	0.21 ^b^	0.00 ^b^	0.63 ^a^	0.00 ^b^	0.12	0.002	1.000	0.203

^1^ Values are least-squares means of 10 replicates unless otherwise stated.^2^ N CON = non-challenged (naïve) control; P CON = challenged control; SAL = salinomycin at 60 mg/kg; EO60 = encapsulated essential oil at 60 mg/kg, EO120 = encapsulated essential oil at 120 mg/kg.^3^ L = linear; Q = quadratic.^4^ SEM = pooled standard errors of the means.

**Table 5:** Effect of dietary encapsulated essential oils on concentrations (mM/g) of cecal volatile fatty acids (VFA) in coccidiosis-vaccine-challenged broiler chickens^1.^

Item	N CON ^3^	P CON	SAL	EO60	EO120	SEM ^5^	*P*-value		
							ANOVA	L ^4^	Q ^4^
Acetate	23.5 ^a^	22.4 ^a^	19.5 ^ab^	21.6 ^a^	16.2 ^b^	1.47	0.010	0.009	0.238
Propionate	2.12	2.08	1.68	2.00	1.36	0.28	0.283	0.077	0.425
Isobutyrate	0.10	0.09	0.08	0.09	0.09	0.01	0.501	0.766	0.699
Butyrate	6.42	7.04	8.74	7.97	5.90	0.82	0.206	0.405	0.210
Isovalerate	0.22	0.30	0.26	0.20	0.21	0.02	0.086	0.028	0.146
Valerate	0.40	0.44	0.40	0.38	0.33	0.04	0.317	0.052	0.925
Lactate	3.01	2.92	2.67	2.80	2.77	0.27	0.951	0.688	0.889
BCFA ^2^	0.73	0.82	0.74	0.67	0.63	0.05	0.102	0.020	0.481
Total SCFA ^2^	35.8 ^a^	35.2 ^a^	33.4 ^ab^	35.0 ^a^	26.9 ^b^	2.11	0.027	0.021	0.189

^1^ Values are least-squares means of 10 replicates unless otherwise stated. ^2^ BCFA = branched-chain fatty acid (isobutyrate + valerate + isovalerate; total SCFA = total short-chain fatty acid (acetate + propionate + butyrate + isobutyrate + valerate + isovalerate). ^3^ N CON = nonchallenged (naïve) control; P CON = challenged control; SAL = salinomycin at 60 mg/kg; EO60 = encapsulated essential oil at 60 mg/kg, EO120 = encapsulated essential oil at 120 mg/kg. ^4^ L = linear; Q = quadratic. ^5^ SEM = pooled standard errors of the means. ^abc^ Means without a common superscript letter differ (*P* < 0.05).

**Table 6:** Effect of dietary encapsulated essential oils on body surface temperature (°C) in coccidiosis-vaccine-challenged broiler chickens ^1.^

Item	N CON ^2^	P CON	SAL	EO60	EO120	SEM ^4^	*P*-value		
							ANOVA	L ^3^	Q ^3^
Day 28									
Head	35.6 ^a^	36.1 ^a^	35.8 ^a^	33.9 ^b^	32.4 ^c^	0.26	<.0001	<.0001	0.374
Breast	35.2 ^ab^	35.3 ^a^	35.6 ^a^	34.0 ^bc^	33.5 ^c^	0.40	0.002	0.002	0.448
Leg	36.4 ^a^	37.0 ^a^	36.7 ^a^	35.2 ^b^	34.6 ^b^	0.38	<.0001	0.000	0.206

^1^ Values are least-squares means of 10 replicates unless otherwise stated. ^2^ N CON = nonchallenged (naïve) control; P CON = challenged control; SAL = salinomycin at 60 mg/kg; EO60 = encapsulated essential oil at 60 mg/kg, EO120 = encapsulated essential oil at 120 mg/kg. ^3^ L = linear; Q = quadratic. ^4^ SEM = pooled standard errors of the means. ^abc^ Means without a common superscript letter differ (*P* < 0.05).

**Table 7:** Effect of supplementation of encapsulated essential oils on serum biochemical parameters in coccidiosis-vaccine-challenged broiler chickens ^1.^

Item ^2^	N CON ^3^	P CON	SAL	EO60	EO120	SEM ^5^	*P*-value		
							ANOVA	L ^4^	Q ^4^
TCHO, mg/dl	111	104	97.7	109	101	4.80	0.287	0.692	0.299
TG, mg/dl	146	151	142	194	144	25.2	0.598	0.828	0.139
GOT, IU/L	170	169	156	160	168	7.92	0.669	0.918	0.467
GPT, IU/L	3.10	3.70	3.40	3.33	3.40	0.29	0.691	0.377	0.476
UA, mg/dl	7.19	8.07	6.96	7.03	6.56	0.53	0.372	0.065	0.692
Nitric oxide, μM	16.8	21.7	14.5	19.3	19.2	2.82	0.500	0.619	0.768

^1^ Values are least-squares means of 10 replicates unless otherwise stated.^2^ TCHO = total cholesterol; TG = triglyceride; GOT = glutamic oxaloacetic transaminase; GPT = glutamic pyruvic transaminase; UA = urea acid. ^3^ N CON = nonchallenged (naïve) control; P CON = challenged control; SAL = salinomycin at 60 mg/kg; EO60 = encapsulated essential oil at 60 mg/kg, EO120 = encapsulated essential oil at 120 mg/kg. ^4^ L = linear; Q = quadratic. ^5^ SEM = pooled standard errors of the means.

**Table 8:** Effect of supplementation of encapsulated essential oils on serum antioxidant parameters in coccidiosis-vaccine-challenged broiler chickens ^1.^

Item ^2^	N CON ^3^	P CON	SAL	EO60	EO120	SEM ^5^	*P*-value		
							ANOVA	L ^4^	Q ^4^
MDA, μM/L	12.6 ^b^	25.2 ^a^	27.8 ^a^	21.1 ^ab^	30.6 ^a^	3.67	0.012	0.369	0.201
Catalase, U/mL	21.0	11.4	14.9	15.1	16.6	3.04	0.310	0.073	0.665
SOD, U/mL	80.0	77.5	69.7	76.3	74.9	3.88	0.431	0.662	0.990
TAC, μM Trolox equivalents	601	633	564	589	553	28.6	0.378	0.361	0.191

^1^ Values are least-squares means of 10 replicates unless otherwise stated. ^2^ MDA = malondialdehyde; SOD = superoxide dismutase; TAC = total antioxidant capacity. ^3^ N CON = nonchallenged (naïve) control; P CON = challenged control; SAL = salinomycin at 60 mg/kg; EO60 = encapsulated essential oil at 60 mg/kg, EO120 = encapsulated essential oil at 120 mg/kg. ^4^ L = linear; Q = quadratic. ^5^ SEM = pooled standard errors of the means.^abc^ Means without a common superscript letter differ (*P* < 0.05).
